# Molecular phylogenetic assessment of *Spirobranchus kraussii*-complex (Annelida: Serpulidae) from the Japanese Archipelago

**DOI:** 10.7717/peerj.11746

**Published:** 2021-07-14

**Authors:** Genki Kobayashi, Ryutaro Goto

**Affiliations:** Seto Marine Biological Laboratory, Field Science Education and Research Center, Kyoto University, Shirahama, Nishimuro, Wakayama, Japan

**Keywords:** Phylogeography, Polychaetes, Ryukyu Islands, Tokara Gap, Species complex

## Abstract

*Spirobranchus kraussii* (Annelida: Serpulidae) was recognized as being widely distributed both in the Pacific and Atlantic Oceans. However, the sampling records far from its type locality (South Africa) have been questioned. Actually, recent molecular phylogenetic studies showed that *S. kraussii* contains genetically distinct species. In this study, we performed molecular phylogenetic analyses of *S.* cf. *kraussii* collected from Japan using the nucleotide sequences of a mitochondrial gene and two nuclear genes. Three lineages were recovered within *Spirobranchus kraussii*-complex in Japan, and one (*Spirobranchus* sp. 6) showed moderate genetic difference (approximately 4%) in the mitochondrial cytb gene sequence from *Spirobranchus* sp. 1, an undescribed sequenced species from Honshu Island, Japan. However, the nucleotide sequences of the 18S rRNA gene and ITS2 region were nearly indistinguishable. The other lineage was clearly distinct from the other previously sequenced species and is thus considered to be another distinct species of this species complex (*Spirobranchus* sp. 5). Although detailed morphological assessment of these lineages is necessary to define their taxonomic status, the present study provided further implications for the species diversity within the *S*.* kraussii*-complex.

## Introduction

Recognizing precise species boundaries is required to soundly conduct applied studies, such as conservation and ecological studies (Mace, 2004). Although distinguishing morphologically similar species has been challenging for biologists (Winker, 2005; Bickford et al., 2007), the use of low-cost DNA sequencing techniques has made it much easier. These techniques are useful for examining widely distributed species that may tend to contain two or more genetically distinct species. Marine organisms have been especially suggested to commonly contain cryptic species (Knowlton, 2000; Nygren, 2014).

The Japanese Archipelago is known to harbor rich species diversity in marine environments, involving many annelid species (Fujikura et al., 2010). The type localities of some Japanese annelids are far away from Japan and thus they have been treated as cosmopolitan species (Imajima & Hartman, 1964; Imajima, 1996). However, recent molecular phylogenetic studies or DNA barcoding on these annelids have provided growing evidence that they contain genetically distinct species, for example, in Capitellidae (Tomioka et al., 2016), Dinophiliidae (Jimi et al., 2020), *Marphysa* spp. (Eunicidae) (Abe et al., 2019), Maldanidae (Kobayashi et al., 2018), Nereididae (Tosuji et al., 2019), and Spionidae (Simon, Sato-Okoshi & Abe, 2019). Many other cosmopolitan annelids remain to be tested by a molecular phylogenetic analysis or DNA barcoding.

**Figure 1:**
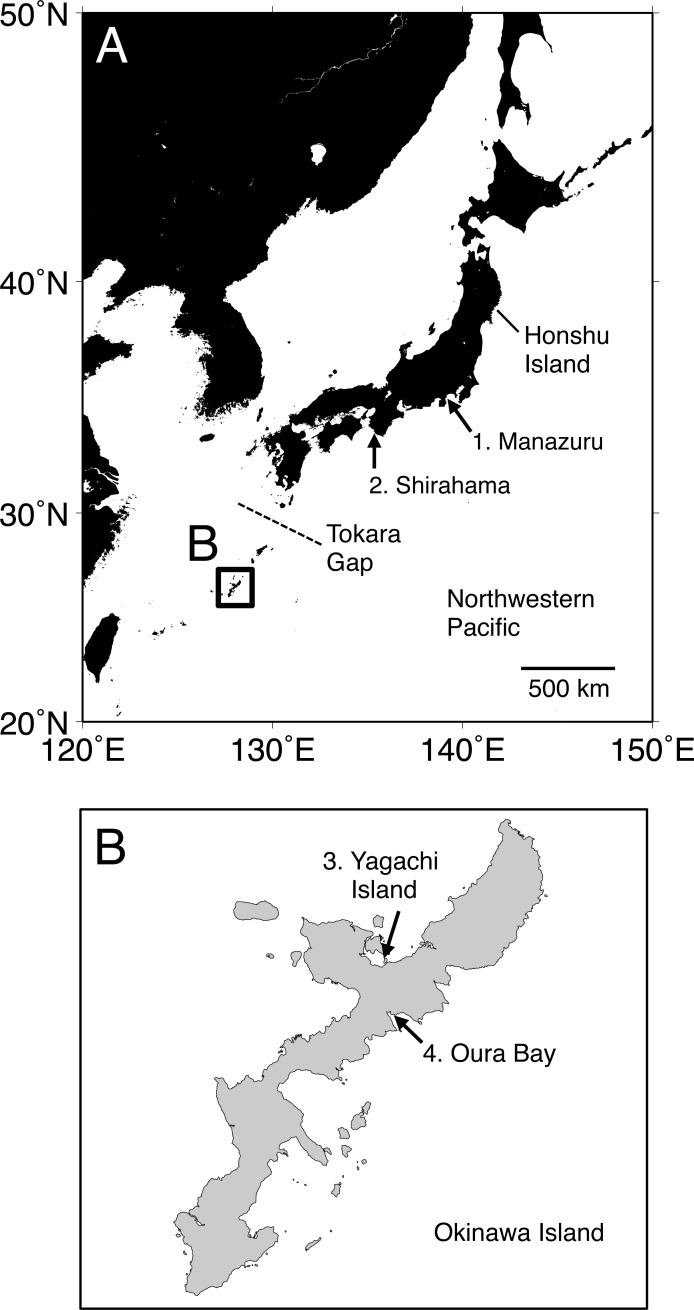
Map of the sampling localities of *Spirobranchus kraussii*-complex in Japan used in the present study. (A) Japan. (B) Okinawa Island. 1. Manazuru, Kanagawa (Simon et al., 2019); 2. Shirahama, Wakayama; 3. Yagachi Island, Okinawa; 4. Oura Bay, Okinawa.

A serpulid species *Spirobranchus kraussii* (Baird, 1865) was recognized as a cosmopolitan species ranging from South Africa to Hawaii, through the Indian Ocean, the Mediterranean, China, and Japan (Pillai, 1971; Imajima, 1996; Fiege & Sun, 1999; Coles & Eldredge, 2002; Çinar, 2006; Belal, 2012), although the records from the areas far from its type locality (South Africa) were questioned (Çinar, 2013; Hutchings & Kupriyanova, 2018). Indeed, *S.* cf. *kraussii* from the Japanese coasts has been suggested to be a different species because the morphology of uncini (comb-shaped chaetae) differs from that of the type specimen(s) of *S*. *kraussii* (Imajima, 1996). Recent molecular phylogenetic analyses in Simon et al. (2019) suggested that only South African specimens are *S*. *kraussii* and those from the other countries, including Manazuru, Honshu Island, Japan (Fig. 1), are different species. Japanese specimens of the *S*. *kraussii*-complex are temporarily referred to as *Spirobranchus* sp. 1 because it is clearly unnamed and undescribed (Simon et al., 2019). *Spirobranchus* sp. 1 has been recorded from a wide range of the coastal area in Japan, that is, Honshu Island to Okinawa Island, Ryukyu Islands, based on morphological observations (Nishi, 1993; Imajima, 1996) (Fig. 1). Intra-specific genetic divergences have been documented for coastal invertebrates between the Honshu and Ryuky Islands (Ogoh & Ohmiya, 2005; Kojima et al., 2006; Kawane, Wada & Watanabe, 2008; Yamada, Furukawa & Wada, 2009; Yamazaki et al., 2017), using the nucleotide sequences of mitochondrial DNA. Considering the background of the genetic differentiation between Honshu and Okinawa Islands, *Spirobranchus* sp. 1 on these islands may include unstudied genetically distinct lineages or species. However, molecular data of *Spirobranchus* sp. 1 are limited to the mitochondrial cytochrome b (cytb) gene and nuclear 18S rRNA gene sequences of specimens collected from Honshu Island (Simon et al., 2019).

In the present study, we conducted phylogenetic analyses of *Spirobranchus* cf. *kraussii* from Japan based on the nucleotide sequences of mitochondrial genes and nuclear DNA to reveal their species diversity. Morphological differences between each genetically distinct lineage were also mentioned and discussed.

## Materials & Methods

Specimens were collected at Shirahama, Wakayama (33°41′38″N, 135°20′17″E), and Oura Bay (26°33′10″N, 128°02′24″E) and Yagachi Island (26°39′01″N, 128°01′46″E), Okinawa Island (Fig. 1). The specimens are deposited in Seto Marine Biological Laboratory (Shirahama, SMBL-V0604–V0611; Oura Bay, SMBL-V0620–V0627; Yagachi Island, SMBL-V0612–V0619). The map of the sampling locations was produced using Generic Mapping Tools (GMT) v5.1.1. (Wessel et al., 2013). All specimens were fixed and preserved in 99% ethanol because we planned to use the specimens for population genetics. Morphology of the specimens was examined and photographed under a stereomicroscope. Terms of serpulid morphology follows Ten & Kupriyanova (2009) and “flap” indicates a part of tube that is flat projection of keel over the tube entrance (Ten & Kupriyanova, 2009; Sun, Hove & Qiu, 2012; Simon et al., 2019). Portions of the body wall of the specimens were cut out and treated with a mixture of 9 µL of proteinase K solution (Nacalai Tesque, Kyoto, Japan) and 100 µL of 10% solution of Chelex 100 Resin (Bio-Rad, Hercules, CA) at 56 °C for >30 min and then at 100 °C for >20 min, and the supernatant fluid was used as template DNA (following Kobayashi et al., 2021).

The partial sequences of the mitochondrial cytb gene, nuclear 18S rRNA gene, and internal transcribed spacer-2 (ITS2) region were determined (following Kobayashi et al., 2021). The PCR mixtures were as follows: (1) 8.75 µL of sterilized water, 0.06 µL of TaKaRa Ex *Taq* Hot Start Version (TaKaRa Bio, Kusatsu, Japan), 1.25 µL of 10 × Ex *Taq* Buffer, 1.0 µL of 2.5 µM dNTP mixture, 0.15 µL of 20 µM forward and reverse primers for the mitochondrial cytb gene (cytbF/cobr825; Table 1) or the nuclear ITS2 region (ITS3/ITS4; Table 1), and 1.0 µL of template DNA; or (2) KOD One PCR Master Mix (TOYOBO, Osaka, Japan) (18SA1/1800r; Table 1), and 1.0 µL of template DNA. PCR amplifications were performed as follows: (1) initial denaturation at 94 °C for 120 s; followed by 35 cycles of denaturation at 94 °C for 30 s, annealing at 50 °C for 40 s, and extension at 72 °C for 20 s; and then a final extension at 72 °C for 300 s (TaKaRa Ex *Taq*) or (2) 30 cycles comprising denaturation at 98 °C for 10 s, annealing at 60 °C for 5 s, and extension at 68 °C for 2 s (KOD One PCR Master Mix). The PCR products were purified using ExoSAP-IT (Thermo Fisher Scientific, Waltham, MA). Sequencing was outsourced to Eurofins Genomics (Tokyo, Japan). The obtained nucleotide sequences were deposited in the DNA Data Bank of Japan (DDBJ) with DDBJ/EMBL/GenBank accession number LC604687–LC604692 and LC625520–LC625537 (cytb), LC604685–LC604686 (18S), and LC604679–LC604684 (ITS2). Nucleotide sequences of the cytb gene were translated into amino acid sequences using the invertebrate mitochondrial genetic code with MEGA v7.0.26 (Kumar, Stecher & Tamura, 2016) to examine amino acid substitutions among lineages of Japanese *Spirobranchus*.

**Table 1:** Nucleotide sequences of the primers used in this study.

Gene/region	Primer	Sequence (5′–3′)	Direction	Usage^a^	Reference
18S	18A1	CCTACCTGGTTGATCCTGCCAG	Forward	P	Steiner & Dreyer (2003)
	NS2	GGCTGCTGGCACCAGACTTGC	Reverse	S	White et al. (1990)
	NS5	AACTTAAAGGAATTGACGGAAG	Forward	S	White et al. (1990)
	189r	TCGGAATTAACCAGACAAATC	Reverse	S	Nakamura et al. (2007)
	1800r	ATGATCCTTCCGCAGGTTCACC	Reverse	P	Steiner & Dreyer (2003)
ITS2	ITS3	GCATCGATGAAGAACGCAGC	Forward	P/S	White et al. (1990)
	ITS4	TCCTCCGCTTATTGATATGC	Reverse	P/S	White et al. (1990)
cytb	CytbF (aka Cytb424F)	GGWTAYGTWYTWCCWTGRGGWCARAT	Forward	P/S	Boore & Brown (2000)
	cobr825	AARTAYCAYTCYGGYTTRATRTG (I was replaced with Y)	Reverse	P/S	Burnette, Struck & Halanych (2005)

**Notes.**

a P: PCR, S: sequencing.

Phylogenetic analysis based on the concatenated gene sequences (cytb + 18S rRNA) was conducted using *Galeolaria hystrix* (outgroup) and 15 sequences of *Spirobranchus* species, which were selected according to the report of Pazoki et al. (2020) (Table 2). All sequences, except for our specimens, were obtained from GenBank. In addition, ITS2 phylogeny was reconstructed using 18 sequences of *Spirobranchus* spp. (Table 2). The alignment was performed using MAFFT v7.294b (Katoh & Standley, 2013). The best-fit substitution models were selected based on the AICC using PartitionFinder v2.1.1. (Lanfear et al., 2017): HKY+I+G for the cytb dataset, GTR+I for the 18S rRNA dataset, and TVM+G (replaced with GTR+G in MrBayes) for the ITS2 dataset.

**Table 2:** Species used in present analyses with DDBJ/EMBL/GenBank accession numbers and habitat information. Newly obtained sequences are indicated in bold.

Taxon	Locality	cytb	18S	ITS2
*Spirobranchus aloni*	Israel	MF319301	MF319276	MF319230, MF319232
*Spirobranchus cariniferus*	New Zealand	JX144878	JX144817	–
*Spirobranchus corniculatus*	Israel	MF319311	MF319281	MF319244, MF319254
	Philippines	KP892811	KP892778	KP892792, KP892793
	Australia	KP892795	KP892774	KP892782, KP892783
*Spirobranchus gardineri*	Israel	MF319337	MF319297	MF319262, MF319266
*Spirobranchus kraussii*	South Africa	MK308650	MK308665	–
*Spirobranchus* sp. 1 (sensu Simon et al., 2019)	Manazuru, Japan	MK308653	MK308668	–
	Shirahama, Japan	**LC604687, LC604688,LC625526–LC625531**	–	**LC604683,LC604684**
*Spirobranchus* sp. 2 (sensu Simon et al., 2019)	Hawaii, USA	MK308655	MK308670	-
*Spirobranchus* sp. 3 (sensu Simon et al., 2019)	Australia	MK308647	MK308662	–
*Spirobranchus* sp. 5 (sensu Kobayashi & Goto, 2021; this study)	Yagachi Island, Okinawa Island, Japan	**LC604689, LC604690, LC625520 –LC625525**	**LC604685**	**LC604681, LC604682**
*Spirobranchus* sp. 6 (sensu Kobayashi & Goto, 2021; this study)	Oura Bay, Okinawa Island, Japan	**LC604691, LC604692,LC625532 –LC625537**	**LC604686**	**LC604679,LC604680**
*Spirobranchus latiscapus*	New Zealand	JX144879	JX144821	–
*Spirobranchus sinuspersicus*	Iran	MN372436	MN372443	-
*Spirobranchus* cf. *tetraceros*	Israel	MF319335	MF319295	MF319257, MF319258
*Galeolaria hystrix*	New Zealand	JX144861	JX144799	–

Molecular phylogenetic analyses using the concatenated sequences were conducted using Bayesian inference and maximum likelihood (ML) methods. Bayesian analysis was performed using MrBayes v3.1.2. (Ronquist & Huelsenbeck, 2003). Two parallel runs were performed for 5,000,000 generations (with a sampling frequency of 1,000), using the default value of four Markov chains. The initial 25% of samples were discarded and the subsequent 75% were accepted to ensure that the four chains reached stationary distributions, according to the average standard deviation of split frequencies (Ronquist & Huelsenbeck, 2003). An ML phylogenetic analysis was conducted with IQ-TREE v1.6.12 (Nguyen et al., 2014) using 1000 ultrafast bootstrap replicates. Bayesian analyses were also performed for individual genes with 5,000,000 generations (with a sampling frequency of 1,000) and burn-in at 1,250. The resultant tree was edited using FigTree v1.4.3 (https://tree.bio.ed.ac.uk/software/figtree/). Haplotype networks were constructed with the TCS algorithm (Clement et al., 2002) using the cytb haplotypes of *Spirobranchus* spp. with PopART (Leigh & Bryant, 2015) to visualize the relationships among the haplotypes.

## Results

**Figure 2:**
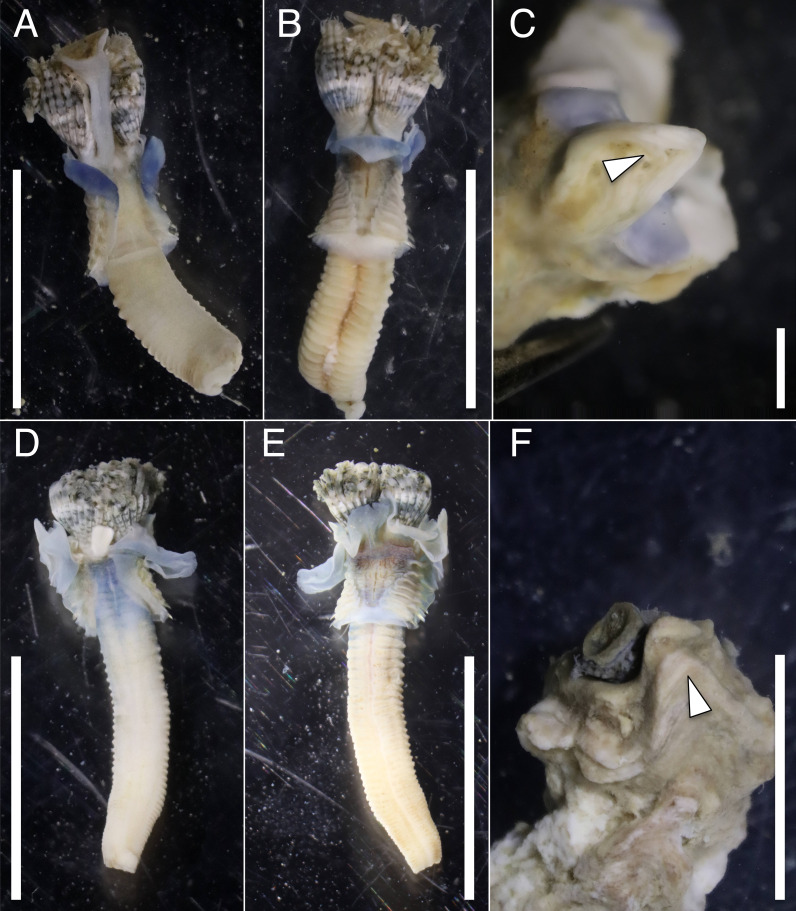
Ethanol fixed specimens of *Spirobranchus* spp. collected from Okinawa Island. * Spirobranchus* sp. 6 from Oura Bay: (A) dorsal view. (B) ventral view. (C) tube. *Spirobranchus* sp. 5 from Yagachi Island: (D) dorsal view (lacking operculum and peduncle). (E) ventral view. (F) tube. Tubes are not derived from the specimens of the body photographs. Arrowheads indicate the flaps overhanging the tube opening. Scale bars = 5 mm (A, B, D, E, F) or 1 mm (C).

**Figure 3:**
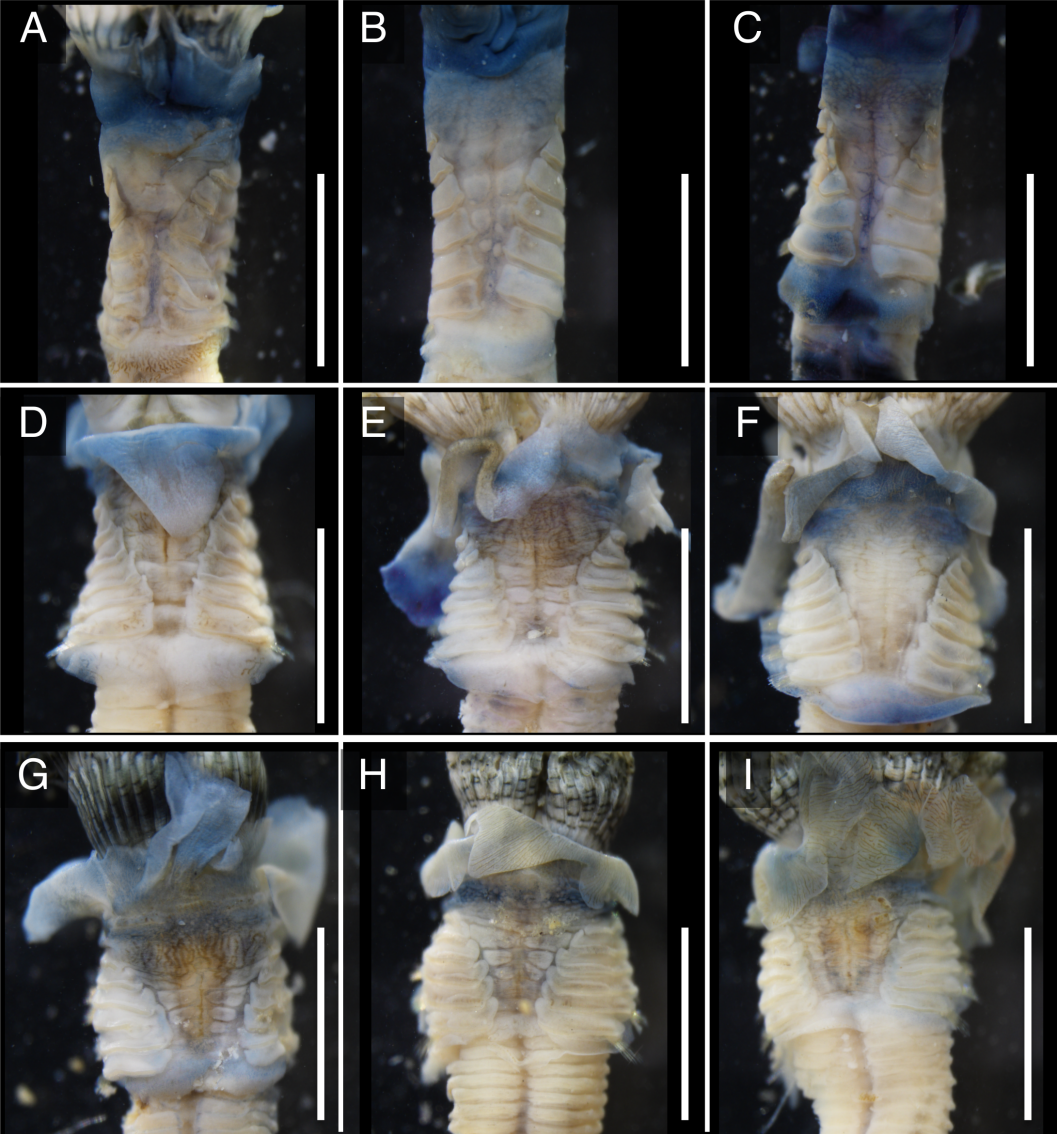
Variations in the thorax of ethanol fixed specimens of Japanese *Spirobranchus* cf. *kraussii*. *Spirobranchus* sp. 1 from Shirahama, Honshu Island: (A) SMBL-V0607, (B) SMBL-V0608; (C), SMBL-V0609. Specimens were extracted from the dwelling tubes after fixation. *Spirobranchus* sp. 6 from Oura Bay, Okinawa Island: (D) SMBL-V0625, (E) SMBL-V0626, (F) SMBL-V0627. *Spirobranchus* sp. 5 from Yagachi, Okinawa Island: (G) SMBL-V0612, (H) SMBL-V0613, (I) SMBL-V0617. Scale bars = 5 mm.

**Figure 4:**
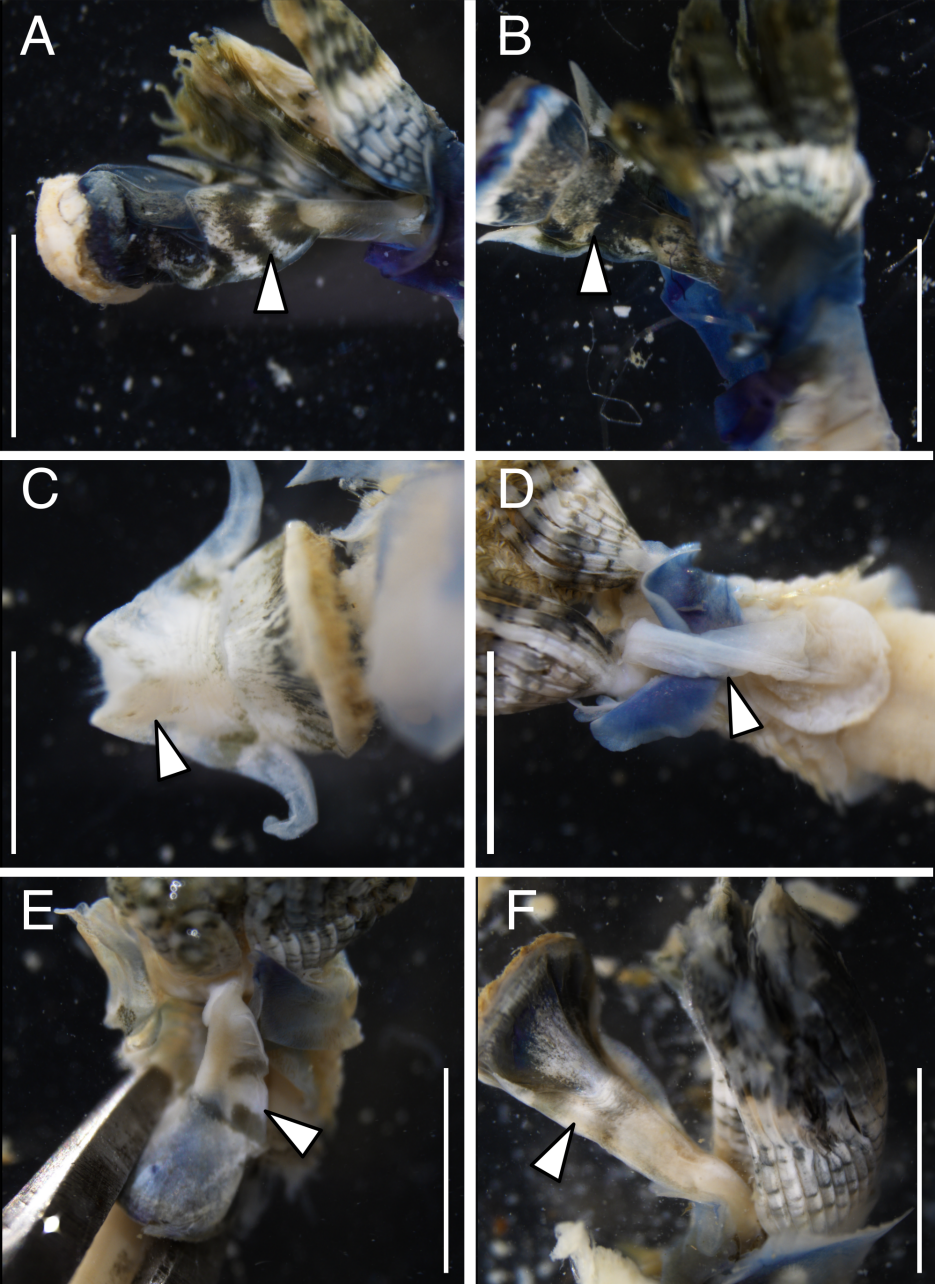
Variation in the color of inner surface of peduncles of ethanol fixed specimen of Japanese *Spirobranchus* cf. *kraussii*. *Spirobranchus* sp. 1 from Shirahama, Honshu Island: (A) SMBL-V0609, (B) SMBL-V0608. *Spirobranchus* sp. 6 from Oura Bay, Okinawa Island: (C) SMBL-V0620, (D) SMBL-V0625. The stem of the peduncle in (C) is missing. *Spirobranchus* sp. 5 from Yagachi, Okinawa Island: (E) SMBL-V0617, (F) SMBL-V0618. Arrowheads indicate the peduncles. Scale bars = 5 mm.

The anterior regions of the specimens collected from Okinawa Island in ethanol were pale blue in color (Fig. 2). A flap-like structure was present at the opening of their dwelling tubes (Figs. 2C and 2F). The collars of the ethanol fixed specimens of Shirahama were blueish, whereas those of Oura and Yagachi were less colored (Fig. 3). The shape of chaetigers and surface morphology in thorax show variations between specimens in each locality (Fig. 3), although this should be carefully examined in the future because the shapes of some specimens were modified due to extraction from tubes after fixation. The ventral surface of peduncles of ethanol fixed specimens differ in color between localities (Fig. 4). Specimens from Shirahama possess a dark-colored peduncle (*n* = 6) with variations in the density of pigmentation between specimens (Figs. 4A and 4B). The peduncles of specimens collected from Okinawa Islands were rather whitish and never heavily pigmented like the specimens of Shirahama (Figs. 4C–4F). Specimens at Oura lack conspicuous pigmented bands (*n* = 5) (Figs. 4C and 4D). Four specimens at Ygachi possessed lightly pigmented bands while one specimen lacks bands on the peduncle (*n* = 5) (Figs. 4E and 4F).

**Figure 5:**
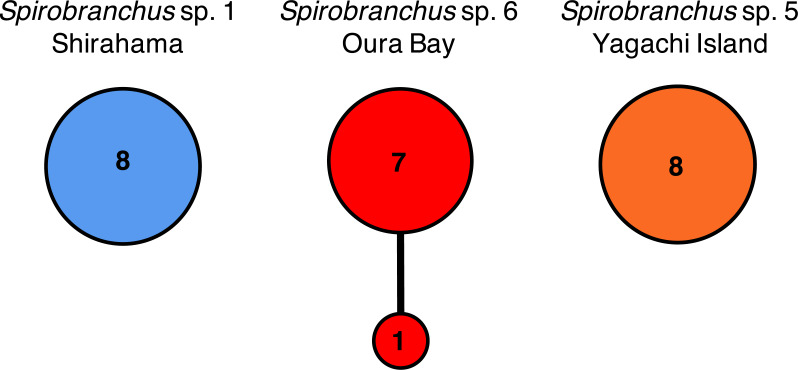
Haplotype networks of *Spirobranchus* cf. *kraussii* in the Japanese Archipelago based on the partial nucleotide sequences of the mitochondrial cytb gene. *Spirobranchus* sp. 1 at Shirahama (356 bp), *Spirobranchus* sp. 6 at Oura Bay, Okinawa Island (356 bp), *Spirobranchus* sp. 5 from Yagachi, Okinawa Island (349 bp). A line indicates a single nucleotide substitution. Numbers in the circles indicates the frequency of the specimens.

Nucleotide sequences of the cytb gene were identical within Shirahama (356 bp, *n* = 8) and Yagachi (349 bp, *n* = 8), while the two haplotypes were found at Oura Bay (356 bp, *n* = 8) (Fig. 5). In addition, the cytb sequences were identical among specimens collected from two localities on Honshu Island, that is, Manazuru (MK308653) and Shirahama. In contrast, there were differences in the cytb gene sequences of specimens from Honshu Island and two localities in Okinawa Island: approximately 4.0% (14 bp; unique nucleotide substitution for this pair occurred at six sites) between Honshu Island and Oura Bay; approximately 22.1% (77 bp) between Honshu Island and Yagachi; and approximately 22.1% (77 bp) between Oura Bay and Yagachi (Table 3). Two amino acid positions of cytb varied among specimens in Honshu Island and Oura Bay: isoleucine (I)/valine (V) and threonine (T)/serine (S). There were 16 of 116 amino acid substitutions between the specimens collected from Honshu Island and Yagachi Islands. The partial sequences of the 18S rRNA gene (1,612 bp) were identical between the specimens from Oura Bay and Manazuru (MK308668), whereas 17 bp were different between the specimens from Oura Bay (1,840 bp) and Yagachi (1,837 bp) (Table 3). The partial sequences of the ITS2 region of *Spirobranchus* specimens collected from Honshu Island and Oura Bay were 567 bp long, of which only three sites were variable, without indels. In contrast, 52 indels were observed between specimens from Shirahama and Yagachi (611 bp) after alignment.

**Figure 6:**
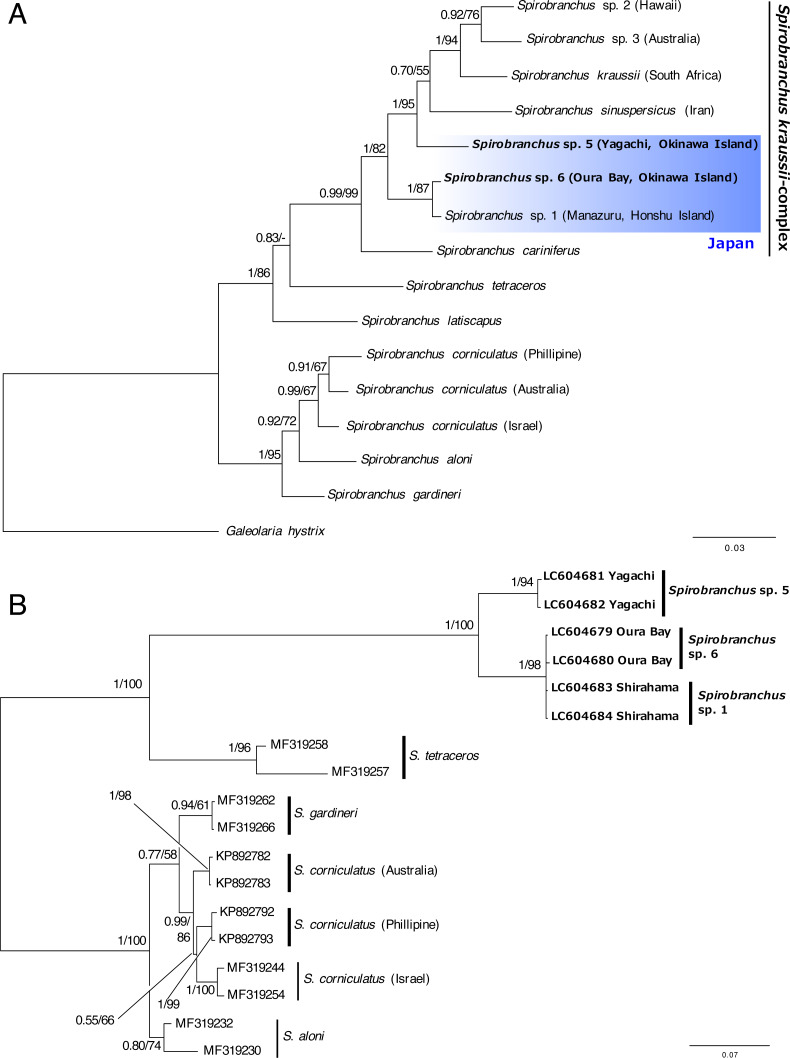
Bayesian phylogenetic trees (A) based on concatenated dataset (mitochondrial cytb + nuclear 18S rRNA gene sequences) and (B) the ITS2 region of the species of Spirobranchus. The numbers above the branches indicate posterior probability, followed by percentage of maximum likelihood bootstrap values above 50% (a hyphen represents <50%). Operational taxonomic units with newly obtained DNA sequences are shown in bold.

The concatenated data set for phylogenetic analysis comprised 2,333 characters of mitochondrial cytb (444 characters) and nuclear 18S rRNA genes (1,889 characters). In the combined data set, 403 sites were variable. Bayesian and ML analyses, using the concatenated dataset yielded the same tree topologies. Therefore, only the Bayesian tree is shown with posterior probabilities (PP) and ML bootstrap values (BS) (Fig. 6A). A specimen of *Spirobranchus* collected from Oura Bay, Okinawa Island, was a sister to *Spirobranchus* sp. 1 collected from Manazuru, Honshu Island (PP = 1, BS = 87%). A specimen of *Spirobranchus* collected from Yagachi Island, Okinawa, was a sister to a cluster that includes all the remaining species of *Spirobranchus kraussii*-complex (PP = 1, BS = 95%).

Bayesian phylogenetic analysis based on the ITS2 sequences revealed that all the species obtained from GenBank were recovered as monophyletic with high support values (PP =1, BS >94%), except for *S. gardineri* (PP = 0.94, BS = 61%) or *S*. *aloni* (PP = 0.80, BS = 74%) (Fig. 6B). The specimens of *Spirobranchus* collected from Shirahama and Oura Bay were monophyletic and phylogenetically indistinct (Fig. 6B). Phylogenetic analyses on the position of the specimen collected from Yagachi Island based on independent genes (cytb and 18S rRNA) resulted largely in low support values, while the specimens from Oura Bay and Honshu Island were clustered in both analyses (Fig. S1).

**Table 3:** Pairwise differences in nucleotide sequences of *Spirobranchus* spp.1, 5, and 6 in Japan. Differences in the nucleotide sequences of 18S rRNA gene (of 1611 bp for Honshu Island and Oura Bay, and of 1837 bp for Oura Bay and Yagachi Island; upper diagonal) and cytb gene (of 349 bp; lower diagonal).

	Honshu Island	Oura Bay	Yagachi Island
Honshu Island (*Spirobranchus* sp. 1)	–	0	0
Oura Bay, Okinawa (*Spirobranchus* sp. 6)	4% (14 bp)	–	0.9% (17 bp)
Yagachi Island, Okinawa (*Spirobranchus* sp. 5)	22.1% (77 bp)	22.1% (77 bp)	–

## Discussion

Recently, the hidden diversity of *Spirobranchus kraussii*- complex was illustrated using molecular analysis (Simon et al., 2019). In addition to *Spirobranchus* sp. 1, which was collected from Honshu Island, Japan (Simon et al., 2019), our phylogenetic analyses revealed two newly identified lineages in *S. kraussii*-complex from Okinawa Island, Japan.

We tentatively referred to *Spirobranchus* from Yagachi Island as *Spirobranchus* sp. 5 because this lineage is clearly genetically distinct from the other lineages (Fig. 6A). As *Spirobranchus* sp. 4 (sensu Simon et al., 2019) has been described as *Spirobranchus sinuspersicus* by Pazoki et al. (2020)*,* we used “sp. 5” for this lineage to avoid confusion. *Spirobranchus* sp. 5 was not clustered with *Spirobranchus* sp. 1 or sp. 6, in the phylogenetic analysis based on the cytb and 18S gene sequences. Although *Spirobranchus* sp. 5 was clustered with the clade of *Spirobranchus* sp. 1 and sp. 6 in the ITS analysis, this is probably because the analysis did not include any other species of *Spirobranchus kraussii*-complex. *Spirobranchus* sp. 5 on Yagachi Island was previously identified as the same species as *S*. *kraussii* based on morphological characteristics (Nishi, 1993) presumably because *S*. *kraussii* was widely believed to be a cosmopolitan species at that time.

The sequence obtained from a specimen of *Spirobranchus* collected from Oura Bay, Okinawa Island, was recovered as a sister to *Spirobranchus* sp. 1, but showed 4% difference in the partial sequence of the cytb gene with *Spirobranchus* sp. 1 on Honshu Island. The Tokara Strait between the northern Ryukyus (the Osumi Islands) and central Ryukyus (Amami-Oshima and Okinawa Islands) is known as a genetic boundary for coastal organisms; both inter- and intra-specific genetic divergence occurred between individuals on Honshu and Ryukyu Islands (Kojima et al., 2003; Kojima et al., 2006; Ogoh & Ohmiya, 2005; Kawane, Wada & Watanabe, 2008; Yamada, Furukawa & Wada, 2009; Wong, Chan & Shih, 2010; Yamazaki et al., 2017). Intra-specific genetic differentiation in the nucleotide sequences of mitochondrial DNA between Honshu and Ryukyu Islands is smaller than *Spirobranchus* in at least three coastal invertebrates, such as: intertidal crabs *Deiratonotus japonicus* (1% in COI) and *Gaetice depressus* (1% in COI)(Kawane, Wada & Watanabe, 2008), and trochid gastropod *Monodonta* spp. (e.g., <1% in COI of *Monodonta labilo* from Wakayama and Iheya Island, Okinawa; LC316230 and LC316267) (Yamazaki et al., 2017). By contrast, the differentiation is larger in an intertidal crab *Ilyoplax pusilla* (9–11% in COI) (Yamada, Furukawa & Wada, 2009), a luminous marine ostracod *Vargula hilgendorfii* (e.g., 8% in cytb between specimens collected from Wakayama and Okinawa Island; AB192865 and AB192726)(Ogoh & Ohmiya, 2005); tideland snails *Cerithidea* spp. (Kojima et al., 2006). In addition, geographic distributions of some genetically close species, probably sister species, are separated between Honshu and Okinawa Islands (Kojima et al., 2003; Wong, Chan & Shih, 2010). The genetic difference is 1.7–2.8% in COI of intertidal snails *Batillaria multiformis* and *Batillaria flectosiphonata* (Kojima, pers. comm.) but 4.4% in COI of *Scopimera globosa* (AB515320) and *Scopimera ryukyuensis* (AB515318) (Wong, Chan & Shih, 2010). The genetic differences in the mitochondrial genes between Honshu and Ryukyu Islands vary between species thus the universal scale for intra- vs. inter-specific variations in the genetic differences between Honshu and Ryukyu Islands is hard to estimate. The genetic difference between Honshu and Oura Bay is quite large, considering the genetic difference of the specimens within Honshu Island (0%; Manazuru and Shirahama) or low genetic diversity at each locality (Fig. 5). The low intra-specific diversity in the cytb gene is common for serpulids, for example, the Mean Tamura Nei pairwise distance of the three species of *Hydroides* were quite low (0%) except for *Hydroides amri* (10%) (Sun et al., 2016).

Although collar and thorax show variations between specimens in each lineage (Fig. 3), the color of the ventral surface of peducles showed variation between the lineages of Japanese *Spirobranchus* cf. *krausii* (Fig. 4). The variation of color in pedancles within species have been reported for *S*. *kraussii* complex (Simon et al., 2019). The difference in the color of peduncles between each locality might be one of the diagnostic characters of these Japanese *Spirobranchus* cf. *krausii*, although the color variation should be examined based on more specimens collected from various localities. Considering both the results on phylogenetic analyses and morphological observation, we tentatively refer to *Spirobranchus* in Oura Bay as *Spirobranchus* sp. 6.

The nuclear ITS2 region has been used to distinguish species of *Spirobranchus* because each clade corresponds to the species identified by morphological observations (Perry et al., 2018). However, in this study, ITS2 analysis did not show phylogenetic differences among specimens collected from Shirahama and Oura Bay, contrary to the results of cytb analysis. The nucleotide sequences of the ITS2 region showed that only 3 of 567 sites were variable. The absence of difference in the nucleotide sequence of the nuclear ITS2 region suggests that either interbreeding still exists between the lineages in Shirahama and Oura Bay, or that the lineage sorting of the two lineages is incomplete. Sun et al. (2016) found two lineages in the cytb phylogeny of an Australian serpulid species, *Hydroides amri*, however, their nucleotide sequences of the ITS2 region were identical. The inconsistency in the genetic differentiation of cytb and ITS corroborate the studies, which showed higher rates of evolution in the mitochondrial genes than the ITS region of marine invertebrates (Blouin, 2002; Vilas, Criscione & Blouin, 2005).

Since the presence of distinct species of *S*. *kraussii* has been suspected for decades (Imajima, 1996; Çinar, 2013; Hutchings & Kupriyanova, 2018), “*S*. *kraussii*” from Australia, Hawaii, Iran and Japan were shown to be different species (Simon et al., 2019; Pazoki et al., 2020). However, the Mediterranean and Red Sea (Shalla & Holt, 1999; Belal, 2012) and Asian (Pillai, 1971; Becker, 1993) specimens of *S*. cf. *kraussii* are yet to be examined. *Spirobranchus* cf. *kraussii* has been recorded from the southern Chinese coast (Tan & Morton, 1998; Fiege & Sun, 1999; Zheng et al., 2004; Sun, Hove & Qiu, 2012; Lin et al., 2017), which is geographically relatively close to Okinawa Island. However, individuals inhabiting China have not been sequenced yet; thus, it is not clear whether Japanese *Spirobranchus* spp. are the same species as Chinese *S*. cf. *kraussii*. Molecular data are needed to reveal whether species in Japanese Archipelago also inhabits mainland Asia or not in the future.

## Conclusions

The present study revealed the genetic diversity of *Spirobrachus kraussii*-complex in Okinawa Island, Japan. Two lineages were found using molecular phylogenetic analysis for the first time. One of them is clearly distinct from the other previously sequenced lineages and is considered to be another distinct species of the complex. Although morphological studies are needed to clarify the taxonomic status of these two lineages, the present study provides further knowledge on the diversity of *Spirobranchus kraussii*-complex.

##  Supplemental Information

10.7717/peerj.11746/supp-1 Supplemental Information 1 Bayesian phylogeny (A) based on mitochondrial gene sequences and (B) 18S rRNA gene sequences of the species of *Spirobranchus* The numbers above the branches indicate posterior probability. Operational taxonomic units with newly obtained DNA sequences are shown in bold.

10.7717/peerj.11746/supp-2 Supplemental Information 2 DNA sequences

## Additional Information and Declarations

### Field Study Permissions

The following information was supplied relating to field study approvals (i.e., approving body and any reference numbers): Permissions are not required in the sampling localities in the present study.

### DNA Deposition

The following information was supplied regarding the deposition of DNA sequences: The nucleotide sequences are available in the DNA Data Bank of Japan (DDBJ) with DDBJ/EMBL/GenBank: LC604687 –LC604692 and LC625520 –LC625537 (cytb), LC604685 –LC604686 (18S), and LC604679 –LC604684 (ITS2).

### Data Availability

The following information was supplied regarding data availability: The raw sequence data are available in a Supplementary File.
