# Supplementary figures and images for: Molecular phylogenetic assessment of Spirobranchus kraussii-complex (Annelida: Serpulidae) from the Japanese Archipelago
Source: PeerJ. 2021 Jul 14;9:e11746. doi: 10.7717/peerj.11746 (PMC8286061; doi:10.7717/peerj.11746)

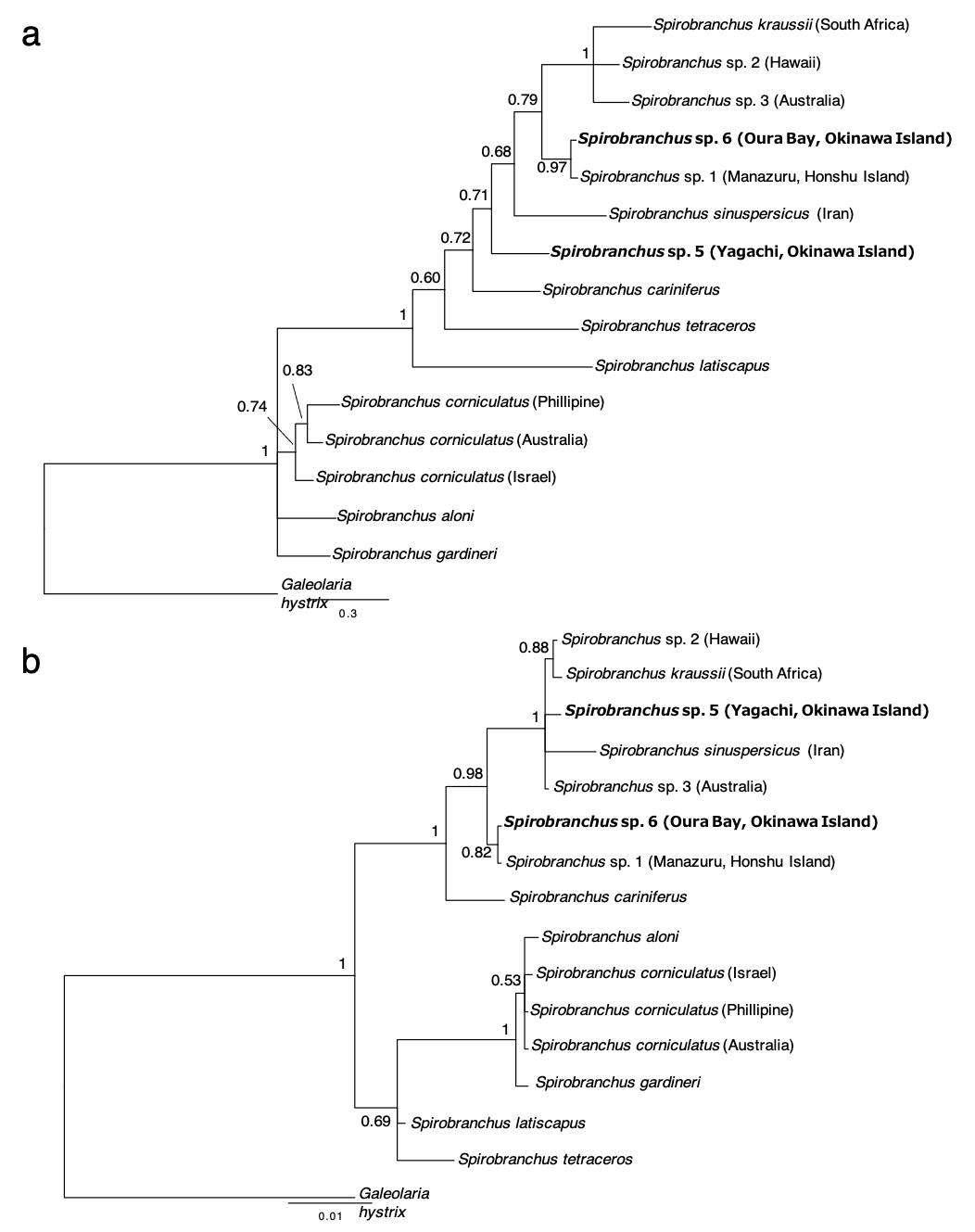

Supplement: Supplemental Information 1 — The numbers above the branches indicate posterior probability. Operational taxonomic units with newly obtained DNA sequences are shown in bold. [file peerj-09-11746-s001.png]
